# Baseline MRI-Radiomics Can Predict Overall Survival in Non-Endemic EBV-Related Nasopharyngeal Carcinoma Patients

**DOI:** 10.3390/cancers12102958

**Published:** 2020-10-13

**Authors:** Marco Bologna, Valentina Corino, Giuseppina Calareso, Chiara Tenconi, Salvatore Alfieri, Nicola Alessandro Iacovelli, Anna Cavallo, Stefano Cavalieri, Laura Locati, Paolo Bossi, Domenico Attilio Romanello, Rossana Ingargiola, Tiziana Rancati, Emanuele Pignoli, Silvana Sdao, Mattia Pecorilla, Nadia Facchinetti, Annalisa Trama, Lisa Licitra, Luca Mainardi, Ester Orlandi

**Affiliations:** 1 Department of Electronics, Information and Bioengineering (DEIB) Politecnico di Milano, 20133 Milan, Italy; valentina.corino@polimi.it (V.C.); luca.mainardi@polimi.it (L.M.); 2 Department of Radiology, Fondazione IRCCS Istituto Nazionale dei Tumori, 20133 Milan, Italy; giuseppina.calareso@istitutotumori.mi.it (G.C.); silvana.sdao@istitutotumori.mi.it (S.S.); 3 Department of Oncology and Hemato-Oncology, Università degli studi di Milano, 20133 Milan, Italy; chiara.tenconi@istitutotumori.mi.it (C.T.); lisa.licitra@istitutotumori.mi.it (L.L.); 4 Medical Physics Unit, Fondazione IRCCS Istituto Nazionale dei Tumori, 20133 Milan, Italy; anna.cavallo@istitutotumori.mi.it (A.C.); domenicoattilio.romanello@istitutotumori.mi.it (D.A.R.); rossana.ingargiola@istitutotumori.mi.it (R.I.); emanuele.pignoli@istitutotumori.mi.it (E.P.); 5 Head and Neck Cancer Medical Oncology 3 Department, Fondazione IRCCS Istituto Nazionale dei Tumori di Milano, 20133 Milan, Italy; salvatore.alfieri@istitutotumori.mi.it (S.A.); stefano.cavalieri@istitutotumori.mi.it (S.C.); laura.locati@istitutotumori.mi.it (L.L.); 6 Radiotherapy 2 Unit, Fondazione IRCCS Istituto Nazionale dei Tumori di Milano, 20133 Milan, Italy; nicolaalessandro.iacovelli@istitutotumori.mi.it (N.A.I.); nadia.facchinetti@istitutotumori.mi.it (N.F.); ester.orlandi@istitutotumori.mi.it (E.O.); 7 Department of Medical and Surgical Specialties, Radiological Sciences and Public Health, University of Brescia, ASST Spedali Civili, 25123 Brescia, Italy; paolo.bossi@unibs.it; 8 Prostate Cancer Program, Fondazione IRCCS Istituto Nazionale dei Tumori, 20133 Milan, Italy; tiziana.rancati@istitutotumori.mi.it; 9 Post-Graduate School in Radiodiagnostics, Università degli Studi di Milano, 20133 Milan, Italy; mattia.pecorilla@unimi.it; 10 Research Department, Fondazione IRCCS Istituto Nazionale dei Tumori di Milano, 20133 Milan, Italy; annalisa.trama@istitutotumori.mi.it

**Keywords:** nasopharyngeal carcinoma, radiomics, magnetic resonance imaging, survival models, EBV-related nasopharyngeal carcinoma

## Abstract

**Simple Summary:**

The prognostic performance of traditional methodologies in advanced nasopharyngeal carcinoma does not allow to successfully stratify patients. Previous studies showed that MRI-radiomics has been used to give additional information to improve the prognosis for this type of pathology in patients from endemic areas (Asia). The purpose of this study was to use MRI-radiomics to develop prognostic models for overall survival in patients from non-endemic areas (Europe or United States). In particular, T1-weighted and T2-weighted MRI were used for the purpose. Radiomic features from those images allowed to successfully train a prognostic signature that improved the prognostic performance of models based on clinical variables alone for different clinical endpoints (overall survival, disease-free survival and loco-regional recurrence-free survival). These results suggest how MRI-radiomics is a useful additional tool for prognosis in nasopharyngeal cancer.

**Abstract:**

Advanced stage nasopharyngeal cancer (NPC) shows highly variable treatment outcomes, suggesting the need for independent prognostic factors. This study aims at developing a magnetic resonance imaging (MRI)-based radiomic signature as a prognostic marker for different clinical endpoints in NPC patients from non-endemic areas. A total 136 patients with advanced NPC and available MRI imaging (T1-weighted and T2-weighted) were selected. For each patient, 2144 radiomic features were extracted from the main tumor and largest lymph node. A multivariate Cox regression model was trained on a subset of features to obtain a radiomic signature for overall survival (OS), which was also applied for the prognosis of other clinical endpoints. Validation was performed using 10-fold cross-validation. The added prognostic value of the radiomic features to clinical features and volume was also evaluated. The radiomics-based signature had good prognostic power for OS and loco-regional recurrence-free survival (LRFS), with C-index of 0.68 and 0.72, respectively. In all the cases, the addition of radiomics to clinical features improved the prognostic performance. Radiomic features can provide independent prognostic information in NPC patients from non-endemic areas.

## 1. Introduction

Nasopharyngeal carcinoma (NPC) is a malignancy with a distinct geographical distribution worldwide, commonly affecting Asian countries (incidence rate, IR, up to 20–50 per 100.000 persons/year) and rarely European countries’ populations (IR of 0.47 per 100.000 persons/year) [1]. In non-endemic areas, all clinical information is commonly translated from locations where NPC is an endemic disease. Despite this, preliminary results from a large multicentric database on NPC patients in non-endemic area showed that survival was comparable to patients in endemic countries [2]. Worldwide, advances in NPC management including intensity-modulated radiotherapy (IMRT) techniques and intensified chemotherapy approaches (induction and concurrent) have contributed to an improved outcome with a lowered frequency of serious radiation-induced toxicities [3,4].

Even in the IMRT era, risk assessment and therapeutic choice for NPC are primarily driven by the tumor-node-metastasis (TNM) staging system [5]. However, highly variable treatment outcomes have been reported in patients who were diagnosed at the same clinical stages [6], suggesting the need of finding further independent factors by exploring intrinsic biological heterogeneity. In the recent years, multi-omics technologies, including radiomics, have been used for characterization and prognosis of many types of cancers [7]. Radiomics is the automatic extraction of a large number of quantitative features from medical images. The final aim is selecting a set of significant characteristics that are able to give overall information about phenotype and microenvironment of the tumor and its response to treatment [8]. Head and neck magnetic resonance imaging (MRI) is the modality of choice for loco-regionally staging of NPC [9]. Recently MRI-based radiomics signatures turned out to significantly predict response to induction chemotherapy and survival in advanced NPC [10,11,12,13,14].

However, whether radiomics signatures could assist in predicting outcome in non-endemic areas and/or it could be a potential tool for increasing the precision of TNM criteria in addition to well-known prognostic biomarkers and baseline Epstein-Barr virus-DNA (EBV-DNA) load has been poorly studied [15].

The first aim of this study was to train an MRI-based radiomic signature as prognostic factor for overall survival (OS) in loco-regionally advanced EBV-related non-endemic NPC patients. The second aim was to assess the applicability of the same signature to the prognosis of other clinical endpoints like disease-free survival (DFS), loco-regional recurrence-free survival (LRFS) and distant metastasis-free survival (DMFS). The last aim was to evaluate the incremental value of the radiomic features to the traditional clinical (TNM staging system), demographical (age, gender) and biological (plasma EBV-DNA viral load) risk factors for the prognosis of the aforementioned clinical endpoints.

## 2. Results

### 2.1. Patients Characteristics

A total of 215 patients were acquired in IRCCS Istituto Nazionale dei Tumori (Milan, Italy) in the selected timeframe (2004–2017). Of these, 136 patients met the inclusion criteria (Section 4.1). In particular, 16 patients were excluded because of lack of follow-up data, 14 patients were excluded due to lack of imaging, 12 were excluded due to lack of EBV evaluation, 23 were excluded because they were EBV-negative, and 14 were excluded because of N0. Detailed clinical biological and treatment data are reported in Table 1. Details about image acquisition parameters and instrumentation are reported in Table 2. Median follow-up was 60 months (interquartile range, 45–64 months). Among the 136 patients, 16 (12%) died and 37 (27%) experienced treatment failure during the follow-up period. Among recurrent patients, there were 8 isolated local failures (22%), 6 isolated regional failures (16%), 3 combined loco-regional failures (8%), 15 distant failures alone (41%), and 5 distant failures with local, regional or loco-regional recurrences (13%).

### 2.2. Features Selection and Survival Models Training

The survival models were trained on the entire dataset of 136 patients using the workflow described in Section 4.9.

In total, 530 stable radiomic features were selected (first features selection step). Of those, 67 were non-redundant (second features selection step). The full list of stable and non-redundant features, together with some descriptive statistics, is available in Tables S1 and S2 of the Supplementary Materials. The optimal selected features set was made of two radiomic features (T-T1w-waveletLLH-firstorder-median and T-T1w-LLL-firstorder-Mean). The T-T1w-waveletLLH-firstorder-median is the median intensity inside the tumor region of interest (ROI) in the waveletLLH transform (low pass filter in x and y directions, high pass filter in z direction) of the T1-weighted (T1w) images. T-T1w-LLL-firstorder-Mean is the mean of the distribution of the grey values inside the tumor ROI in the waveletLLL transform (a low pass filtered image) of T1w images. More details about the radiomic feature selection are available in Section 4.9 and in the Supplementary Materials (Table S3, Figure S1). Table 3 shows the values of some main statistics for each radiomic feature used in the model, before and after the Z-score normalization.

For the clinical model, the selected feature set included age (*p* = 0.0002) and overall TNM VIII stage (*p* = 0.047), which were the only significant and independent prognostic factors for OS among the ones considered (see Section 4.11). Table 4 displays information on the Cox coefficients for those prognostic features in the four trained prognostic models (radiomic, volume-based for the primary tumor, clinical and clinical-radiomic). Between the volume-based models, only the one using the primary tumor volume alone is shown. Data about the model using two volumes (main tumor and primary lymph node) are displayed in the Supplementary Materials, Table S4.

### 2.3. Models Validation and Comparison

To evaluate the unbiased performance of the models, 10-fold cross-validation was used. It is important to note that, in each iteration, a slightly different model was used, so in general, features and coefficients differ from the ones presented in Table 4, but the workflow used is the same. For a full description of the 10 temporary radiomic models used within the 10 cross-validation iterations, refer to Table S5 of the Supplementary Materials. For the volume-based model, only the results of the model related to the primary tumor are displayed. The explorative model based on the volumes of both primary tumor and largest lymph node, which performed worse, is displayed in the Supplementary Materials (Figure S2, Table S6).

**Figure 1:**
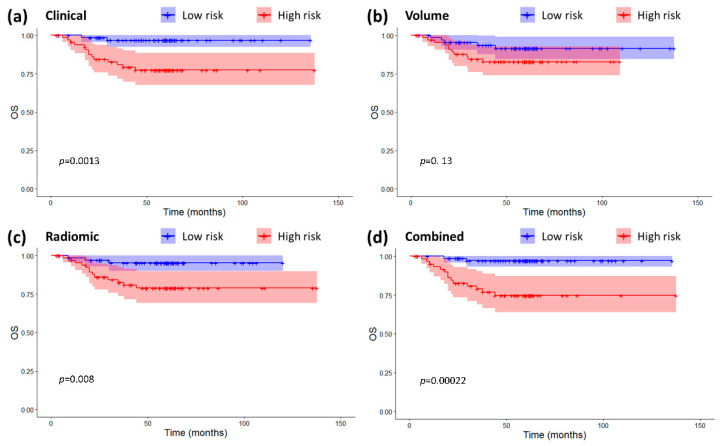
Kaplan-Meier curves and confidence intervals for overall survival (OS) in the high and low risk groups as defined by the four prognostic models. (**a**) Clinical model. (**b**) Volume-based model. (**c**) Radiomic model. (**d**) Combined radiomic-clinical model. The *p*-values for the log-rank tests are also displayed on the plots. Crosses represent censored data.

In terms of prognostic power for OS, the radiomic signature was moderately prognostic (Hazard-Ratio 1.35 95% Confidence Interval (0.98–1.87), *p* = 0.07, C-index 0.68 (0.53–0.82)), the clinical and combined signatures were significantly prognostic (Clinical: HR 1.25 (1.06–1.48), *p* = 0.009, C-index 0.78 (0.72–0.85); Combined: HR 1.61 (1.26–2.07), *p* = 0.0002, C-index 0.83 (0.75–0.91)), and volume was not (HR 0.87 (0.06–13.13), *p* = 0.92, C-index 0.43 (0.32–0.54)). Similar results could be found by looking at the Kaplan-Meier curves for OS of the high/low risk groups (Figure 1). All the binary classifications were significant prognostic factors except for tumor volume (Clinical: HR 7.82 (1.78–34.41), log-rank *p* = 0.0013; Volume: HR 2.23 (0.77–6.41), log-rank *p* = 0.13; Radiomic: HR 4.68 (1.33–16.41), log-rank *p* = 0.008; Combined: HR 9.72 (2.28–42.76), log-rank *p* = 0.00022).

**Figure 2:**
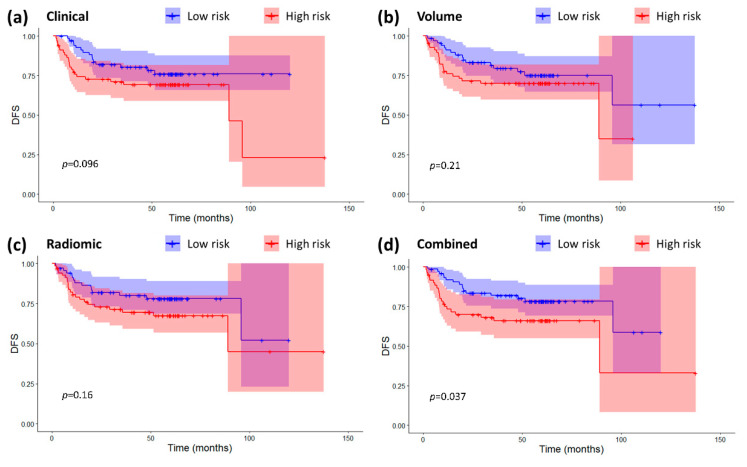
Kaplan-Meier curves and confidence intervals for disease-free survival (DFS) in the high and low risk groups as defined by the four prognostic models. (**a**) Clinical model. (**b**) Volume-based model. (**c**) Radiomic model. (**d**) Combined radiomic-clinical model. The *p*-values for the log-rank. tests are also displayed on the plots. Crosses represent censored data.

In terms of prognostic power for DFS, radiomics and volume were not significant prognostic factors for DFS (Volume: HR 1.16 (0.20–6.72), *p* = 0.87, C-index = 0.54 (0.44–0.64); Radiomic: HR 1.16 (0.92–1.47), *p* = 0.20, C-index 0.60 (0.50–0.70)) while the clinical and combined signature were (Clinical: HR 1.18 (1.02–1.35), *p* = 0.02, C-index = 0.62 (0.53–0.71); Combined: HR 1.27 (1.03–1.56), *p* = 0.02, C-index 0.62 (0.53–0.72)). The high/low risk stratification based on the combined radiomic-clinical model (HR 1.98 (1.03–3.80), log-rank *p* = 0.037) was the only one causing a significant split in the Kaplan-Meier curves (Figure 2). All the other models were not significant even though the clinical classification showed a moderate split between the curves (Clinical: HR 1.73 (0.90–3.44), log-rank *p* = 0.096; Volume: HR 1.52 (0.79–2.92), log-rank *p* = 0.21; Radiomic: HR 1.60 (0.83–3.08), log-rank *p* = 0.16).

**Figure 3:**
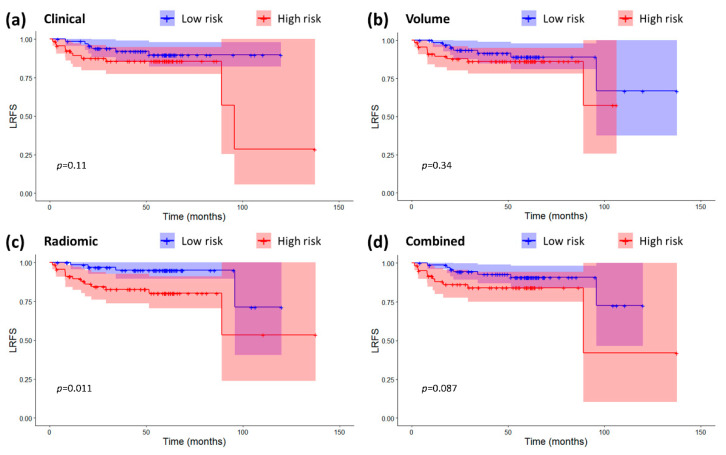
Kaplan-Meier curves and confidence intervals for loco-regional recurrence-free survival (LRFS) in the high and low risk groups as defined by the four prognostic models. (**a**) Clinical model. (**b**) Volume-based model. (**c**) Radiomic model. (**d**) Combined radiomic-clinical model. The *p*-values for the log-rank tests are also displayed on the plots. Crosses represent censored data.

For as far as LRFS is concerned, both the radiomic and the combined signature were prognostic factors (Radiomic: HR 1.69 (1.23–2.32), *p* = 0.001, C-index 0.72 (0.61–0.83); Combined: HR 1.32 (1.10–1.59), *p* = 0.003, C-index 0.66 (0.51–0.81)), the clinical signature was only moderately prognostic (HR 1.32 (0.98–1.80), *p* = 0.07, C-index 0.61 (0.45–0.78)), while tumor volume was not prognostic at all (HR 0.64 (0.04–10.9), *p* = 0.76, C-index 0.47 (0.34–0.59)). In terms of risk classification (Figure 3), only the radiomics-based classification caused a significant split in the Kaplan-Meier curves (HR 3.89 (1.26–11.96), log-rank *p* = 0.011), while the other model did not (Clinical: HR 2.22 (0.82–6.03), log-rank *p* = 0.11; Volume: HR 1.60 (0.61–4.22), log-rank *p* = 0.34; Combined: HR 2.30 (0.86–6.12), log-rank *p* = 0.087).

**Figure 4:**
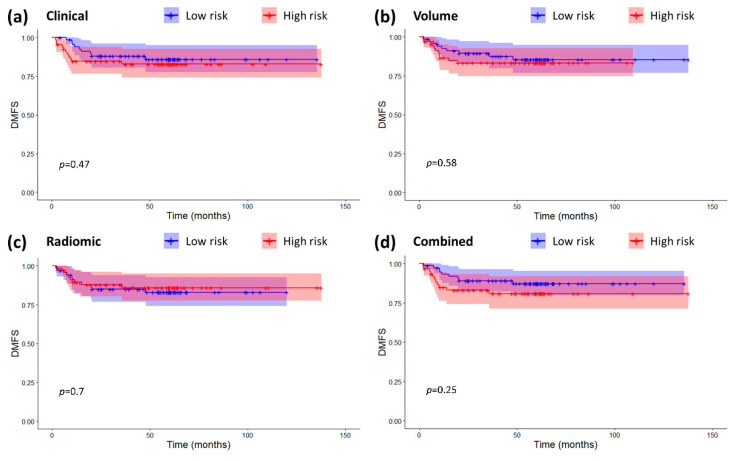
Kaplan-Meier curves and confidence intervals for loco-regional distant metastasis-free survival (DMFS) in the high and low risk groups as defined by the four prognostic models. (**a**) Clinical model. (**b**) Volume-based model. (**c**) Radiomic model. (**d**) Combined radiomic-clinical model. The *p*-values for the log-rank tests are also displayed on the plots. Crosses represent censored data.

No model could provide significant prognostic information for DMFS. This was true for both the continuous signatures (Clinical: HR 1.05 (0.85–1.69), *p* = 0.66, C-index 0.59 (0.46–0.71), Volume: HR 1.73 (0.18–16.89), *p* = 0.64, C-index 0.54 (0.41–0.68); Radiomic: HR 0.92 (0.74–1.15), *p* = 0.46, C-index 0.49 (0.35–0.63); Combined: HR 1.22 (0.92–1.62), *p* = 0.17, C-index 0.62 (0.49–0.75)) and the high/low risk classification (Clinical: HR 1.38 (0.62–3.33), log-rank *p* = 0.47; Volume: HR 1.28 (0.53–3.10), log-rank *p* = 0.58; Radiomic: HR 0.84 (0.35–2.03), log-rank *p* = 0.58; Combined: HR 1.67 (0.69–4.04), log-rank *p* = 0.25). Kaplan-Meier curves for the risk groups are displayed in Figure 4.

### 2.4. Correlation between Radiomic Features and Clinical/Volumetric Variables

**Figure 5:**
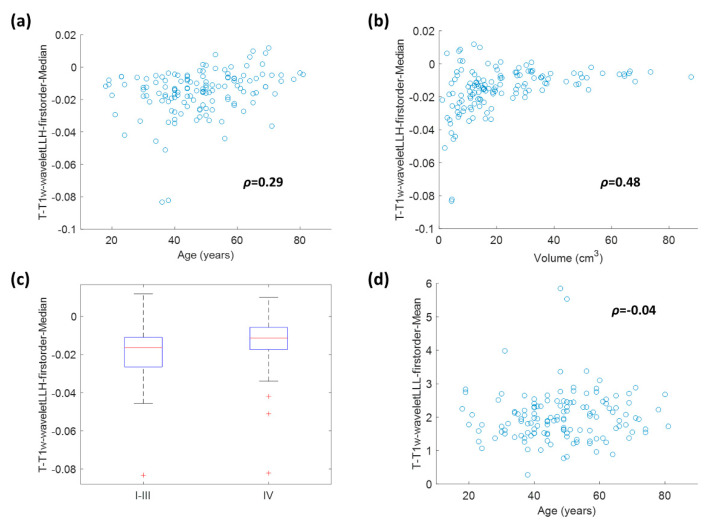
Boxplots and scatterplots representing the association of T-T1w-waveletLLH-firstorder- Median with age (**a**), tumor volume (**b**) and overall stage (**c**), and the association of T-T1w-waveletLLL-firstorder-Mean with age (**d**). The Spearman correlation coefficients are reported on the scatterplots.

The association between the two selected radiomic features with the selected clinical variables (age and overall stage) and tumor volume was also investigated. To do so, Mann-Whitney tests or correlation tests (referred to Spearman correlation coefficient) were used, where necessary. T-T1w-waveletLLH-firstorder-Median was positively correlated with age (ρ = 0.29, *p*-value = 0.006), volume (ρ = 0.48, *p*-value = 1.86 × 10^−8^) and TNM staging (Mann-Whitney *p* = 0.0024). T-T1w-waveletLLL-firstorder-Mean was negatively correlated with age (*p*-value = 2.49 × 10^−6^), but the correlation coefficient was low (ρ = −0.04). A more detailed insight on these significant association is provided by the scatterplots and boxplots in Figure 5.

## 3. Discussion

The main aim of this study was the development of a prognostic signature for OS in EBV-related NPC patients, using radiomic features extracted from the main tumor and the largest lymph node in both T1w and T2w images.

The training pipeline provided a two-feature signature, with both features coming from the main tumor and from T1w images. This does not mean that features from T2w images of from the largest lymph node are completely useless, since in some of the iterations in the training phase some of those features were actually selected as the best (see Table S3), but it just means that, overall, the two selected features are the ones with the best performance. Even though usually radiomic models obtained from multiparametric MRI are the ones that perform better [13], this is not the first time that monomodal radiomic models result to be the best [16]. The results are also partially in contrast with [17], in which the addition of lymph node features significantly improved the results, but this may be explained taking into account the differences in terms of type of pathology (NPC vs. generic head and neck cancer), the definition of the lymphnodal ROI (all the nodal masses vs. the largest lymph node), the different imaging technique (computed tomography vs. MRI).

The cross-validation of the prognostic models showed that the signature based on radiomics alone has significant prognostic power for OS and it was able to distinguish two risk groups with significantly different Kaplan-Meier curves (Figure 1C). Significant divergence in the survival of high and low risk groups were also shown for the clinical (Figure 1A) and combined radiomic-clinical signature (Figure 1D). The combined signature was the one with the best performance. This is in line with the majority of studies of literature on radiomics applications to head and neck cancer (see [10,18] or the training set in [13]).

The performance of the radiomic signature for OS obtained better performance compared to analogous signature developed for the head and neck cancers [8,19]. This may be due to the fact that those models were not trained on a specific area, like the one presented in this study (that was trained on NPC only). The present results were indeed comparable to the ones observed for a prognostic model for OS trained on a larger dataset of computed tomography (CT) images [18]. The performance of the present model was worse than the analogous one developed for patients from endemic areas [10,13] but this may be due to the differences in the type of image used, since those models, unlike ours, also involve contrast-enhanced T1w. It is also interesting to see how a relatively simple model based on Cox regression like the one presented in this study managed to perform similarly, in terms of C-index on the primary endpoint, to a model based on a more complex deep learning model trained on a larger dataset of over 400 patients [20]. When considering the primary endpoint, the C-index of our models (radiomic, clinical or radio-clinical) were in the range 0.68–0.83, while the ones obtained in [20] was in the range 0.69–0.79.

Another aim of the study was the evaluation of the prognostic power of the developed signatures for other clinical endpoints (DFS, LRFS, DMFS). The results showed that the translatability of the signatures was dependent on the particular endpoint with good prognostic power for LRFS and providing an added value for DFS when merged with clinical variables. This is in part due to the fact that the disease-related endpoints are linked to OS and could potentially be used as a surrogate [21,22]. However, the prognostic performance was lower compared to the one measured for OS. This is reasonable when thinking that the radiomic signature is a good but not perfect prognostic factor for OS and that the correlation between OS and other clinical endpoints is not perfect either.

The radiomic signature was the best for prognosis of LRFS. With regard to LRFS, our results are partially in line with those reported in [14], in which the trained radiomic signature was used to predict local recurrence in 737 patients with non-metastatic T4 NPC outperforming clinical prognostic nomogram including age and gross tumor volume (GTV) of primary tumor as defined by a radiation oncologist.

We were not able to find a prognostic role for tumor volume. This is in contrast with the majority of literature [23]. Feng et al. [24] reported that a large GTVT (Gross Tumor Volume of the primary tumor) is a negative prognostic factor for LRFS at 5 years, with a 40 cm^3^ cut-off. Analyzing 321 patients with NPC, Wu et al. [25] found a statistically significant correlation between GTVT and LRFS, DMFS, DFS, and OS (all *p* < 0.05) at univariate and multivariate analyses. In addition, a very recently accepted paper by our team [26] aimed to look into the relationship between IMRT or volumetric modulated arc therapy (VMAT) parameters and 5-year outcome for a consecutive series of non-metastatic NPC patients reported a prognostic value of GTVT on the LRFS trend and identify a volume cut-off of GTVT for the prediction of LRFS at 5 years (43.2 cm^3^). There are differences in terms of patients’ characteristics considered. Indeed, in that paper there were 17.5% of patients staged I-II and 35% of patients in T1 category. Another major difference lies in the way to define GTVT. In all previously mentioned studies concluding that GTVT significantly impacts on the outcome and, in particular, on LRFS, GTVT was defined and outlined by a radiation oncologist on MRI images co-registered with CT ones or only on CT images with or without the help of FDG-PET findings. In this paper, we consider the primary tumor volume as defined by an experienced radiologist only on selected MRI images. This may have influenced the volume measurements. Moreover, the median value of volume, which was used as a cut-off in our study, is much smaller compared to the optimal cut-offs found in the aforementioned studies (16 cm^3^ vs. around 40 cm^3^). As a matter of fact, the value of 40 cm^3^ was the quantile 0.87 of our distribution. This discrepancy between optimal cut-off (from other studies) and median cut-off may be the reason volume does not separate the Kaplan-Meier curves well.

In terms of DFS, the combined radiomic-clinical model (HR 1.98 (1.03–3.80), log-rank *p* = 0.037) was the only one determining a significant split in the Kaplan-Meier curves between low- and high-risk patients. However, The C-index of the combined model in the study was lower than that reported by the previous studies on NPC [10,12,16]. This may depend on the fact that those models were trained specifically for DFS prognosis.

No signature was prognostic for DMFS. In general, all the models had lower prognostic power compared to the one they had for OS. This is reasonable since the models were originally trained to be prognostic for OS. Not only this, but DMFS is considered more relevant to patient N stage, which is determined based on metastatic lymph nodes [27]. However, both the features selected for the prognostic signature for OS (the primary endpoint) came from the primary tumor and therefore capture the spatial heterogeneity of the main tumor alone. Training models specific for other endpoints like DMFS or DFS may lead to the selection of N-related features and to an overall increase in the prognostic performance for DMFS and DFS as well.

Figure 5 shows that there was some correlation between radiomic features and clinical variables, especially for T-T1w-waveletLLH-firstorder-Median. However, these correlations do not make radiomics redundant. As a matter of fact, by looking at Figure 1, Figure 2, Figure 3 and Figure 4, it is possible to see that the combined radiomic-clinical model is always the one with the best performance. This may suggest that, although the radiomic features have significant correlation with clinical variables, they also provide independent prognostic information.

The strength of our study mainly resides in the serious addressing of limitations and biases that can arise in radiomic MRI studies involving images with large heterogeneity in acquisition protocols. Strict pre-processing protocols were used with the aim of establishing a signature possibly robust with respect to heterogeneity biases. A final proof of the generalizability of the here-proposed signature can only be achieved through external independent validations. Post-contrast T1w images were not used for this study, despite being proven a successful source of information [13,28] because, in our case, those type of images were not available for each patient and because they would have added another element of heterogeneity, since both quantity of contrast agents and type of pulse sequence (spin-echo or gradient-echo) may vary.

The main limitation of the study is the absence of an independent validation cohort, and for this reason, internal cross-validation was used to get a first estimate of the model performance on unseen data. Thus, the study can only be classified as a Tripod 2a [29] and may only be considered as explorative. The lack of external validation is due to the fact that collecting an external dataset with sample size comparable to the one of our datasets is a very difficult and time-consuming process, given the rarity of the pathology in non-endemic areas. Previous studies [13,30] split the initial dataset in train and test, but this methodology, although more efficient in providing a unique model to test, reduces the test set size, thus leading to larger confidence intervals in the estimation of the quality metrics and reduced statistical significance of the results. This is particularly critical for OS, in which the number of events, which are fundamentals for the estimation of the quality of a survival model, is very low. For this reason, we preferred to use K-fold cross-validation, which maximizes the test set size, since, throughout the K iterations, all the 136 patients are treated as unseen samples. However, the collection of an independent dataset is still required for the validation of the models that were presented in the results section and is the logical next step of the study. Moreover, since all the data necessary to reproduce the signature have been provided (see Table 3 and Table 4), the validation of the signature could also be done by other research groups, thus leading to a stronger validation of the present work.

Another concern may be raised by the analyses performed to identify the set of stable features. Multiple translations of the ROI were used to assess stability to ROI uncertainties, which is typically assessed by multiple segmentation studies. This surrogate use of ROI translation is of course an approximation because modifications due to multiple segmentations are more random and complex to model. However, different previous studies of literature have shown how techniques based on image or ROI manipulation could be used to perform a preliminary feature selection instead of more traditional test (test-retest or multiple segmentation) with successful results, also in the application to clinical models [31,32,33,34]. For as far as the selection of features that were stable to the variations in the image acquisition parameters is concerned, that was performed based on the results of previous analyses on a virtual phantom representing the brain. Phantoms, although modeled on the anatomy of specific regions of the body, cannot be as accurate as real patients when it comes to assessing stability. However, multiple acquisitions on real patients (except maybe for test-retest) are impractical and, therefore, phantoms are still the best tool available to investigate stability to acquisition-related variability. Although both the aforementioned analyses are only an approximation of the ideal methodology for stability assessment, they can be considered as good trade-off between accuracy of the results and practicality of the analyses. Also, it must be noted that stability is only the first criteria of feature selection and that the further features selection methods will likely exclude potentially undetected unstable features.

Another limitation is that we only included patients with N-positive disease, and this could potentially limit the application of study results to lower disease stage where treatment strategy is also less evidence-based.

## 4. Materials and Methods

### 4.1. Patients Population

The population used for this study was pooled from the patients affected by NPC acquired at Fondazione IRCCS Istituto Nazionale dei Tumori (in Milan, Italy) between 2004 and 2017. Inclusion criteria for the population were the following: (1) availability of clinical and follow-up data; (2) minimum follow-up of 24 months; (3) cancer treatment involving IMRT techniques and chemotherapy (CHT) with or without induction CHT; (4) EBV-encoded RNA (EBER) positivity and availability of EBV-DNA plasma levels, defined as positive or negative; (5) presence of at least 1 pathological lymph node; and (6) availability of pretreatment unenhanced spin-echo T1w and T2w MRI examination data within 45 weeks before any treatment.

EBER status assessment and quantification techniques for EBV-DNA positivity were defined according to our previous work [15].

Staging of the disease was performed with head and neck MRI with and without contrast medium, FDG-PET and/or thorax and abdomen CT scan with and without contrast medium. All patients were restaged according to TNM staging, VIII edition [5].

Only N+ patients were considered for the analysis since it has been shown in previous studies related to head and neck cancer that the addition of radiomic features from the lymphnodal ROI provides a better prognostic ability than radiomics from the main tumor alone [17,35]. Metastatic lymph node involvement was defined according to Ho et al. [36]: criteria included, namely, central necrosis, extra capsular spread, shortest diameter of cervical or medial retropharyngeal lymph nodes >1 cm and >5 mm for lateral retropharyngeal lymph node(s).

Ethical approval by the Ethical Committee of the IRCCS was obtained for this study (INT study number 116/20, date obtained: 19 June 2020).

### 4.2. Treatment

Radiotherapy (RT) was delivered by relying either on a conventional static-field technique (conventional IMRT) or VMAT with sequential or simultaneous integrated boost approaches. IMRT planning have been previously reported [37]. Briefly, GTV including both nasopharyngeal primary tumor (GTVT) and involved lymph nodes (GTVN) as demonstrated by clinical, endoscopic and imaging data (MRI and 18F-FDG PET/CT) was outlined in all patients. To improve the quality of GTV delineation, radiological data (T1w volumetric interpolated breath-hold examination and T2w MRI sequences) were co-registered together with CT planning images and evaluated together with a dedicated head and neck radiologist when necessary. The corresponding planning target volume, consisting of GTVs plus asymmetric margins of 0.5–1.5 cm to account for microscopic diseases and setup uncertainties, was planned to receive a total dose of 70 Gy with conventional or moderately accelerated fractionation (2–2.12 Gy per fraction, 5 fractions per week).

Patients with stage II (T2N1)–III–IV (according to TNM VIII) received concomitant platinum-based CHT. Induction CHT (iCHT) with docetaxel, cisplatin and 5-fluorouracil was added to patients with a potential higher risk of distant metastasis, according to our previously reported institutional policies [37].

### 4.3. Follow-up

After IMRT completion, patients were clinically evaluated at predefined intervals, typically every 3–6 months for the first 3 years and annually thereafter. MRI and 18F-FDG-PET were prescribed on a regular basis and when deemed necessary according to patients’ disease status.

### 4.4. MRI Acquisition

**Figure 6:**
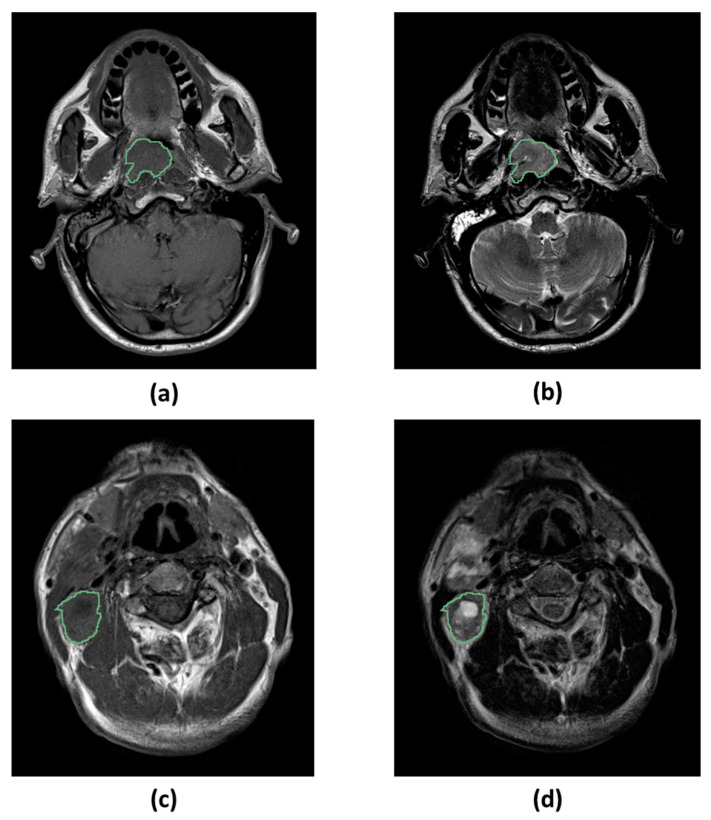
Types of magnetic resonance images and regions of interest used for the study. (**a**) Primary tumor on T1-weighted image. (**b**) Primary tumor on T2-weighted image. (**c**) Largest affected lymph node on T1-weighted image. (**d**) Largest affected lymph node on T2-weighted image. All the images represent 2D slices from a 3D region of interest.

MRI images were acquired for each patient, using different scanners with magnetic field strength of 1.5 T. Unenhanced T1w and T2w MRI were considered, since they are part of the clinical routine. All images were acquired using turbo spin-echo pulse sequence. Other image acquisition parameters were not controlled. Examples of T1w and T2w MRI for a patient with NPC are displayed in Figure 6.

### 4.5. Image Segmentation

An expert radiologist (G.C.) performed manual segmentation of the ROIs, which in this case were the main tumor and the largest lymph node affected by the pathology (Figure 6). Only one segmentation was performed using both the image types (unenhanced T1w and T2w) as a reference to ensure the best result even when the tumor was not visible in T1w due to poor contrast. The segmentation was performed slice by slice by the radiologist, but the final result was a 3D ROI that was used as a mask to extract the radiomic features from both T1w and T2w. This could be done because the images were well registered and the misalignment between corresponding tissues was minimal (Figure 6). Intra-tumoral necrotic and cystic regions at baseline were also included in the ROI since they may be an imaging hint to differentiate different tumor phenotypes. Contrast-enhanced T1w, when available, were not used for the segmentation process, since they may overestimate the size of the lesion in case of edemas spread around the main tumor.

### 4.6. Image Preprocessing

In order to reduce the effect due to the variability of different types of noise, different steps of image preprocessing were applied. Preprocessing of the MRI images included image denoising with a Gaussian filter and a bias field correction using the N4ITK algorithm [38] to correct for potential effects due to inhomogeneity of the magnetic field. Z-score standardization was performed to normalize the MRI signal from different images. Lastly, the images were resampled to a common isotropic resolution of 2 mm (as in [39]) using B-spline interpolation. Intensity values of the MRI were discretized using 32-bins histogram discretization. The preprocessing was performed in MATLAB 2018a (the Mathworks, Natick, MA, USA), using wrappers for other software like 3D Slicer 4.10 [40] or Pyradiomics 2.2.0 [41]. MATLAB was also used for all the other steps of radiomic features extraction and data analysis.

### 4.7. Radiomic Features Extraction

From each image type (T1w or T2w), 536 radiomic features were extracted from both the tumor and the largest affected lymph node, for a total of 2144 features. Since features were extracted with Pyradiomics, they were compatible with the Image Biomarker Standardization Initiative (IBSI)[42]. Features of different categories were considered: 14 shape and size (SS) features, 18 first order statistics (FOS) features, 40 textural features, 24 computed on grey level co-occurrence matrix (GLCM) and 16 computed on grey level run-length matrix (GLRLM). FOS and textural features were also computed for the eight images obtained by the first level wavelet decomposition of the MRI volume. For a more detailed description of the features refer to Pyradiomics documentation [43]. The textural features were limited to the ones obtainable with GLCM and GLRLM, since they are used in most of the study related to radiomics and are available in most of the software and libraries used for radiomic features extraction.

### 4.8. Survival Endpoints

OS and DFS were defined as the time from the first day of treatment (CHT or RT, whichever came first) to death or failure from any cause, respectively. LRFS was the time between the first day of treatment to the occurrence of the first loco-regional recurrence. DMFS was the time between first day of treatment and occurrence of the first distant metastasis. Survival and recurrence time observations were plotted according to the Kaplan-Meier method.

### 4.9. Radiomic Features Postprocessing and Radiomic Model Development

The first postprocessing step of the radiomic features was Z-score normalization, that was performed to ensure comparable ranges for the feature values.

**Figure 7:**
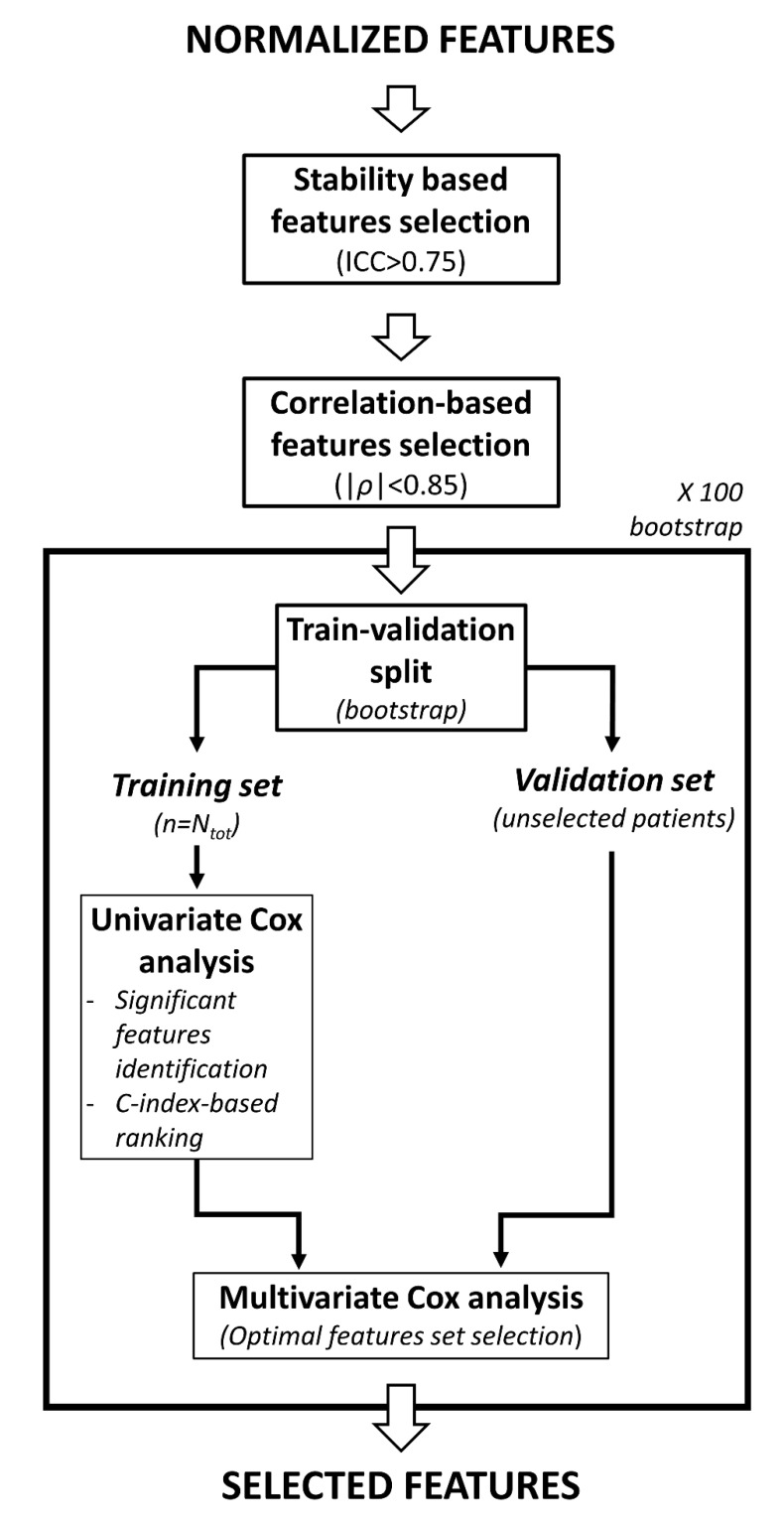
Workflow representing the features selection pipeline used in this study. The N_tot_ in the figure refers to the number of subjects in the entire dataset.

A first selection of radiomic features was based on stability (Figure 7). Only the features that were known to be stable to changes in image acquisition parameters and to geometrical transformation of the ROI were kept. Two experiments to test stability were performed as described in previous studies [31,44]. In the first experiment [44], radiomic features were extracted from multiple virtual MRI acquisitions of the same phantom to assess the stability to variations in image acquisition parameters such as time of repetition (TR), time of echo (TE) and voxel size. In the second experiment [31], a stability analysis to small translation of the ROI was performed as a surrogate of stability to multiple segmentations. In both tests, intra-class correlation coefficient (ICC) was computed to quantify features stability, and radiomic features with ICC > 0.75 in both tests were selected (as defined by [45]).

The second step of features selection was based on feature pairwise correlation (Figure 7) and was performed to ensure a set of features with low internal redundancy. Spearman correlation coefficient ρ was computed for each pair of features and in case a pair had |ρ| > 0.85, only the features with the lower mean Spearman coefficient with all the others was selected.

The next feature selection step was a supervised feature selection method based on univariate and multivariate Cox regression [46] (Figure 7). First, only the features that were significantly associated to survival in univariate analysis (*p* < 0.05 after Benjamini-Hoechberg correction for false discovery rate [47]) were kept. These features were sorted by their Harrel’s C-index [48] and progressively added to a multivariate Cox regression model in order to identify the best feature set (the one that maximized the C-index for the internal validation). This process was repeated 100 times, using bootstrap to define the training set and the internal validation set for each iteration (Figure 7). At the end of the process 100 different features sets were found, each with a different number of features N_i_. The optimal number of features (N_opt_) was selected as the rounded average of the 100 values of N_i_. The N_opt_ features that were chosen the most throughout the 100 iterations were selected for the final radiomic model.

The N_opt_ selected features were used to train a multivariate Cox proportional Hazard regression model and to derive a signature for OS. The radiomic signature was defined as the linear combination of the selected radiomic features and the corresponding regression coefficients. A higher signature value should correspond to a higher risk of death. The median value of the signature in the training set was used as a threshold to classify the patients in high and low risk.

### 4.10. Volume-Based Model Development

Since previous prognostic signatures were found to perform worse than tumor volume [49], another prognostic model based only on the volume of the primary tumor was developed to be used as a reference. The signature was computed, and high/low risk groups were identified according to median signature in the training set. It was chosen to use the volume of the primary tumor because that is a radiomic feature that is very easy to measure and is therefore commonly acquired in the clinical practice and has been used as a reference model in previous analyses [49]. Another volume-based exploratory model using two volumes (the one of the main tumor and the one of the largest lymph node) was also developed.

### 4.11. Clinical Model Development

A model based only on clinical variables was also fitted on the data to be used as reference. The following clinical variables were initially considered for the model: age, sex, overall stage TNM VIII (stage I-III vs. IV), type of treatment (with/without induction) and EBV-DNA plasma load (positive and negative). The same supervised features selection method previously described was used to select the best feature set and a Cox proportional hazard regression model for OS was fitted using the selected features. The signature was computed, and high/low risk groups were identified according to median signature in the training set.

### 4.12. Combined Model Development

A combined radiomic and clinical survival model was developed by using the selected clinical and radiomic features and a Cox proportional hazard regression model for OS was fitted on the resulting feature set. The signature was computed, and high/low risk groups were identified according to median signature in the training set.

### 4.13. Models Validation and Comparison

Since no independent validation dataset was available, internal cross-validation was implemented to provide an estimate of the performance of the models (clinical, volume-based, radiomics and radiomics + clinical) on unseen data. In particular, 10-fold cross-validation was performed. In each iteration, all the parameters (Cox coefficients, mean and standard deviation for Z-score normalization, features to be selected and threshold for high/low risk classification) were estimated on the train set (the first nine folds) using the pipeline described in Section 4.9 and a temporary radiomic model was created. The temporary radiomic model was then applied to compute an unbiased signature and risk class for the instances of the test set (the 10th fold). At the end of the 10 cross-validation iterations, one unbiased signature and label was available for each patient. Those two arrays (signature values and labels) were used to estimate the performance metrics of the models for the different clinical endpoints: OS, DFS, LRFS, DMFS.

First, HR of the cross-validated signature and its significance were evaluated. The second metric used was Harrel’s C-index between the cross-validated signature and the survival. The significance of the association with survival was evaluated through Likelihood ratio test. Another quality metric was the *p*-value of the log-rank test [50] comparing the Kaplan-Meier curves [51] for the high and low risk groups. Last, HR was also computed for the binary risk stratification. The signature evaluation and comparison were performed in R 3.6.1.

The prognostic performance of the cross-validated signatures was evaluated for all the clinical endpoints. We explicitly chose to keep the same signature for DFS and OS since they are associated outcomes. Indeed, DFS was identified as valid surrogate endpoint for OS for early assessment of treatment effects in locally advanced NPC patients [21,22]. Also, prognostic power of LRFS and DMFS was also evaluated as an exploratory analysis.

### 4.14. Correlation between Radiomic Features and Clinical/Volumetric Variables

To improve the interpretability of the radiomic results, proper statistical analyses were performed to evaluate association between the selected radiomic features and the selected clinical variables. Such analyses included either Mann-Whitney test, or computation of Spearman Correlation coefficient. Heatmap with the *p*-values were represented. Significant association between clinical and selected variables were identified and further explored.

## 5. Conclusions

The presented results showed first how MRI-based radiomics from T1w and T2w images can be used to create a prognostic signature for OS in advanced nasopharyngeal cancers from non-endemic areas. This study could potentially be a reference for future radiomics studies in which the training set is made of images acquired with non-standardized protocols, and it could also be useful for the development of future radiomic-based prognostic models, especially for cancers arising from the head and neck area.

## Figures and Tables

**Table 1:** Clinical and treatment characteristics for the patients with EBV-related nasopharyngeal cancers included in the study.

PATIENTS CLINICAL DATA (*N* = 136)	
Age at diagnosis (years) ^1^	48 (39–57)
Sex	Females: 41 (30%) Males: 95 (70%)
T stage (VIII edition)	T2: 77 (57%) T3–T4: 59 (43%)
N stage (VIII edition)	N1–N2: 69 (51%) N3: 67 (49%)
Overall TNM stage (VIII edition)	I–III: 50 (37%) IV: 86 (63%)
EBER positivity	Positive: 136 (100%)
EBV-DNA load	Positive: 122 (90%) Negative: 14 (10%)
Treatment	RT alone: 2 (1%) Concomitant CHT-RT: 43 (32%) Induction CHT + concomitant CHT-RT: 91 (67%)

^1^ Quantitative variables are listed as median and quartiles. CHT: chemotherapy; RT: radiotherapy. EBV: Epstein-Barr Virus; EBER: EBV-encoded RNA.

**Table 2:** Acquisition parameters for the different sequences of magnetic resonance imaging (MRI). Parameters are shown for both T1-weighted and T2-weighted images.

MRI ACQUISITION PARAMETERS		
Image Type	T1-Weighted	T2-Weighted
MRI scanner	Siemens Magnetom Avanto 1.5 T: 133 Others 1.5 T: 3	
Pulse sequence	Spin-echo	
Echo train length ^1^	3 (3–3)	13 (13–13)
Number of averaging ^1^	2 (2–2)	2 (2–2)
Time of repetition (ms) ^1^	524 (477–588)	4670 (3230–5300)
Time of echo (ms) ^1^	12 (12–12)	109 (107–109)
Slice thickness (mm) ^1^	3 (3–3)	3 (3–3)
Slice spacing (mm) ^1^	3.9 (3.9–3.9)	3.9 (3.9–3.9)
Pixel spacing (mm) ^1^	0.57 (0.57–0.69)	0.51 (0.49–0.57)
Flip angle (°) ^1^	127 (127–127)	134 (134–134)
RF coil	Body	

^1^: Quantitative variables are listed as median and quartiles.

**Table 3:** Descriptive statistics for the two radiomic features used in the radiomic model. The values are shown up to the third decimal digit.

RADIOMIC FEATURES STATISTICS		
Feature	T-T1w-WaveletLLH-Firstorder-Median	T-T1w-WaveletLLL-Firstorder-Mean
Mean (before/after normalization)	−0.015/0	2.006/0
Standard deviation (before/after normalization)	0.014/1	0.737/1
Median (before/after normalization)	−0.013/0.151	1.940/−0.090
Interquartile range (before/after normalization)	0.014/1.016	0.841/1.141
10th percentile (before/after normalization)	−0.032/−1.209	1.260/−1.012
90th percentile (before/after normalization)	−0.003/0.918	2.743/1.000

**Table 4:** Regression coefficients for the four Cox proportional hazard regression models (radiomic, volume-based, clinical and combined clinical-radiomic). The coefficients are displayed up to the second decimal digit. For radiomics features, the coefficients are used to multiply the normalized features after Z-score. The threshold to discriminate high and low risk groups and the baseline hazard risk at 60 months are also reported.

COX MODELS COEFFICIENT				
Feature Name	Radiomic Model	Clinical Model	Combined Model	Volume Model
T-T1w-waveletLLH-firstorder-Median	1.11	-	0.69	-
T-T1w-waveletLLL- firstorder-Mean	−0.75	-	−0.45	-
Tumor volume ^1^	-	-	-	9.75 × 10^−6^
Age ^2^	-	0.07	0.05	-
Overall stage (VIII edition)	-	1.48	1.27	-
Threshold for high risk	0.29	4.29	3.23	0.16
Baseline Cumulative hazard (60 months)	0.12	0.12	0.11	0.14

^1^: Volumes are measured in mm^3^. ^2^: Age is measured in years.

## Supplementary Materials

The following are available online at https://www.mdpi.com/2072-6694/12/10/2958/s1, Figure S1: Graph showing the selected features throughout all the 100 bootstrap iterations, Figure S2: Kaplan-Meier curves of the high/low risk groups as defined by the two-volume models, Table S1: Descriptive statistics of the 67 stable and non-redundant features used in the study, Table S2: Descriptive statistics of the 67 stable and non-redundant features used in the study, Table S3: List of stable and non-redundant features ordered by the number of times they were selected within the 100 bootstrap iterations. Table S4: Descriptive statistics and Cox coefficients for the two volumes of interest (main tumor and largest lymph node, Table S5: Radiomic features by iteration of the 10-fold cross-validation, Table S6: Quality metrics with 95% confidence intervals for the two-volumes model (tumor and main lymph node) for the different endpoint of interest.
