# Supplementary material for: Baseline MRI-Radiomics Can Predict Overall Survival in Non-Endemic EBV-Related Nasopharyngeal Carcinoma Patients
Source: Cancers (Basel). 2020 Oct 13;12(10):2958. doi: 10.3390/cancers12102958 (PMC7601980; doi:10.3390/cancers12102958)
Supplement: Supplementary file 1 [file cancers-12-02958-s001.pdf]

Supplementary Material: Baseline MRI-Radiomics Can Predict Overall Survival in Non-Endemic EBV-Related Nasopharyngeal Carcinoma Patients

Marco Bologna, Valentina Corino, Giuseppina Calareso, Chiara Tenconi, Salvatore Alfieri, Nicola Alessandro Iacovelli, Anna Cavallo, Stefano Cavalieri, Laura Locati, Paolo Bossi, Domenico Attilio Romanello, Rossana Ingargiola, Tiziana Rancati, Emanuele Pignoli, Silvana Sdao, Mattia Pecorilla, Nadia Facchinetti, Annalisa Trama, Lisa Licitra, Luca Mainardi and Ester Orlandi

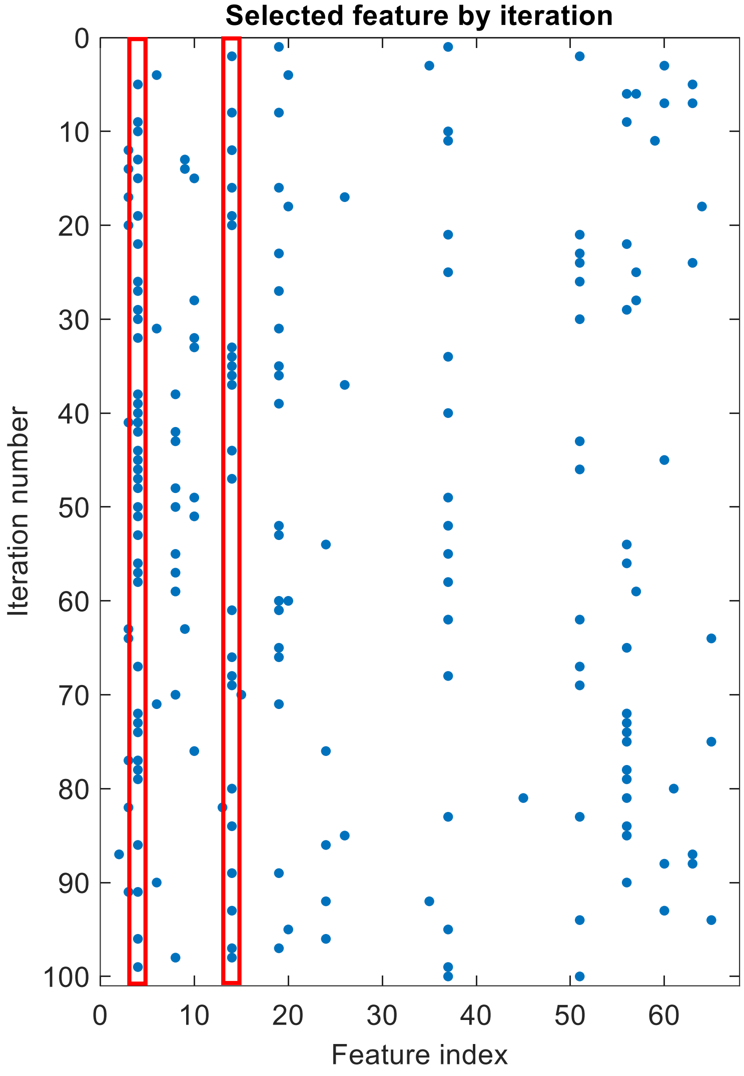

Figure S1. Graph showing the selected features throughout all the 100 bootstrap iterations. Each selected feature is represented by a blue dot and is characterized by its index (on the x-axis) and the iteration in which it was selected (on y-axis). The red rectangles highlight the features selected for the final model: T-T1w-waveletLLH-firstorder-Median (feature 4). which was selected 39 times; T-T1w-waveletLLL-firstorder-Mean (feature 14). which was selected 23 times.

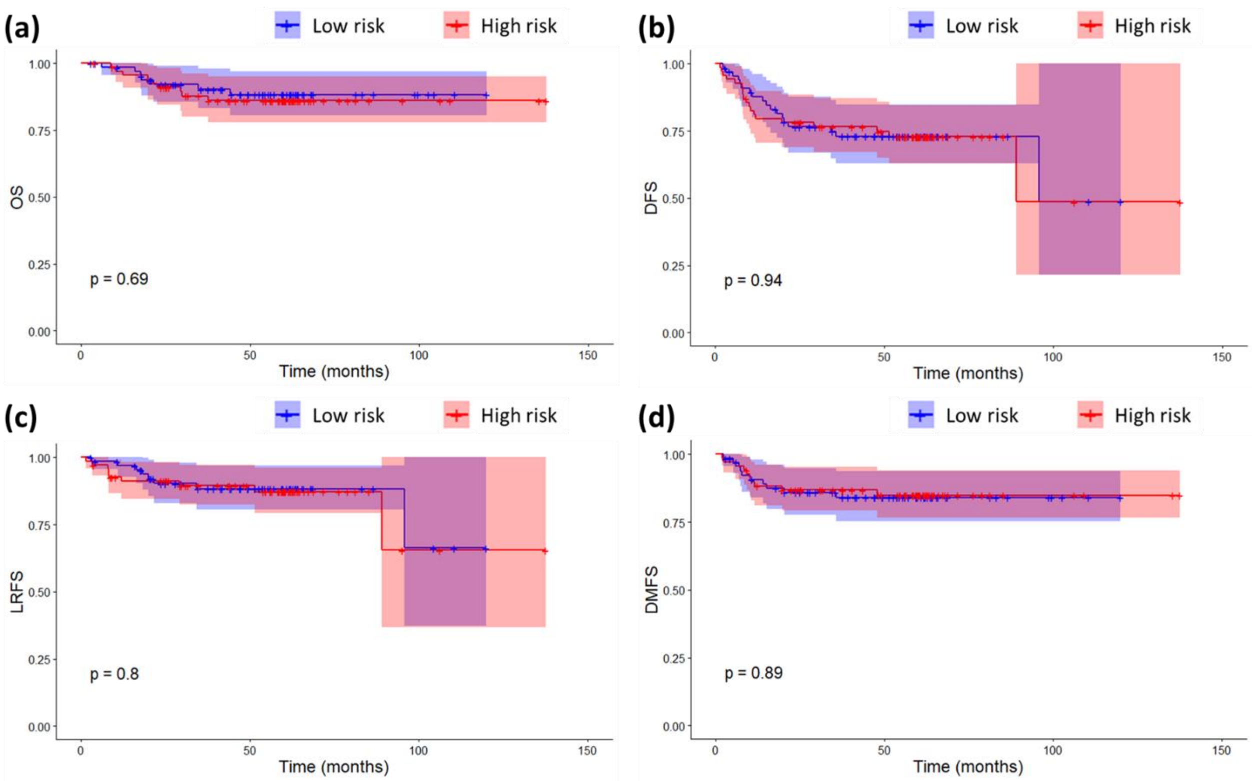

Figure S2. Kaplan-Meier curves of the high/low risk groups as defined by the two-volume models. (**a**) Overall survival (OS). (**b**) Disease-free survival (DFS). (**c**) Locoregional recurrence-free survival (LRFS). (**d**) Distant metastasis-free survival.

Table S1. Descriptive statistics of the 67 stable and non-redundant features used in the study.

Stable_Feat_Name	Mean Value	Std Value	Median Value	Iqr Value	Perc10 Value	Perc90 Value
T_T1w_original_shape_Flatness	0.475	0.133	0.448	0.188	0.324	0.653
T_T1w_original_shape_MajorAxisLength	47.847	11.133	47.053	12.163	35.040	61.171
T_T1w_original_glcm_InverseVariance	0.391	0.041	0.398	0.044	0.327	0.438
T_T1w_waveletLLH_firstorder_Median	−0.015	0.014	−0.013	0.014	−0.032	−0.003
T_T1w_waveletLLH_glcm_JointEntropy	6.094	0.630	6.106	0.873	5.336	6.884
T_T1w_waveletLHL_firstorder_10Percentile	−0.326	0.118	−0.304	0.133	−0.506	−0.199
T_T1w_waveletLHL_firstorder_90Percentile	0.256	0.068	0.247	0.087	0.164	0.346
T_T1w_waveletLHL_firstorder_Median	−0.024	0.025	−0.017	0.022	−0.056	−0.004
T_T1w_waveletLHL_glcm_SumEntropy	4.012	0.417	4.006	0.639	3.497	4.554
T_T1w_waveletHLL_firstorder_Median	−0.009	0.019	−0.009	0.013	−0.024	0.003
T_T1w_waveletHLL_glcm_DifferenceEntropy	2.839	0.325	2.824	0.436	2.462	3.306
T_T1w_waveletHHL_glcm_SumSquares	9.133	5.090	8.103	4.970	4.280	14.854
T_T1w_waveletHHH_glcm_ClusterProminence	1436	1254	1013	1021	473	3093
T_T1w_waveletLLL_firstorder_Mean	2.006	0.737	1.940	0.841	1.260	2.743
T_T1w_waveletLLL_firstorder_RobustMeanAbsoluteDeviation	0.303	0.103	0.290	0.124	0.194	0.411
T_T1w_waveletLLL_firstorder_TotalEnergy	110713	132663	68670	96758	20253	237051
T_T2w_original_shape_Elongation	0.719	0.127	0.715	0.200	0.557	0.891
T_T2w_original_shape_MinorAxisLength	34.149	8.753	33.813	11.325	23.849	45.338
T_T2w_original_firstorder_90Percentile	1.610	0.383	1.583	0.440	1.196	2.050
T_T2w_original_firstorder_MeanAbsoluteDeviation	0.414	0.067	0.407	0.090	0.332	0.506
T_T2w_original_glcm_MaximumProbability	0.027	0.013	0.022	0.019	0.014	0.048
T_T2w_original_glcm_SumEntropy	4.860	0.234	4.874	0.340	4.546	5.184
T_T2w_original_glrlm_LongRunHighGrayLevelEmphasis	310.831	91.383	307.410	129.228	207.622	433.612
T_T2w_waveletLLH_firstorder_Mean	−0.018	0.025	−0.018	0.035	−0.046	0.011

T_T2w_waveletLLH_glcm_ClusterProminence	5377	3662	4283	4374	1840	10188
T_T2w_waveletLLH_glcm_Imc1	−0.118	0.042	−0.111	0.033	−0.163	−0.084
T_T2w_waveletLLH_glcm_MaximumProbability	0.066	0.041	0.054	0.047	0.029	0.120
T_T2w_waveletLHL_glcm_JointEntropy	7.262	0.682	7.372	1.062	6.302	8.081
T_T2w_waveletHLL_glcm_Imc1	−0.107	0.049	−0.094	0.034	−0.164	−0.071
T_T2w_waveletHHL_glcm_Idm	0.279	0.061	0.280	0.090	0.201	0.348
T_T2w_waveletHHH_firstorder_Entropy	3.563	0.396	3.549	0.579	3.126	4.099
T_T2w_waveletLLL_firstorder_Range	9.018	2.067	8.937	3.152	6.462	11.781
T_T2w_waveletLLL_glcm_ClusterShade	−13.056	268.428	−3.136	317.749	−361.580	324.519
N_T1w_original_shape_Elongation	0.608	0.150	0.604	0.205	0.430	0.813
N_T1w_original_shape_Flatness	0.431	0.123	0.418	0.157	0.280	0.599
N_T1w_original_shape_Maximum2DDiameterColumn	43.130	15.179	43.012	22.170	22.457	60.491
N_T1w_original_firstorder_Median	0.731	0.353	0.732	0.416	0.311	1.091
N_T1w_original_glcm_InverseVariance	0.352	0.077	0.376	0.093	0.219	0.428
N_T1w_waveletLLH_firstorder_Mean	0.006	0.019	0.005	0.016	−0.010	0.030
N_T1w_waveletLLH_firstorder_Median	0.011	0.019	0.007	0.016	−0.004	0.035
N_T1w_waveletLLH_glcm_ClusterProminence	7629	8163	4679	7371	1420	21439
N_T1w_waveletLLH_glcm_JointEntropy	6.038	1.213	6.272	1.565	4.071	7.263
N_T1w_waveletLHL_firstorder_10Percentile	−0.374	0.198	−0.318	0.295	−0.648	−0.164
N_T1w_waveletLHL_firstorder_Median	0.024	0.045	0.013	0.031	−0.005	0.074
N_T1w_waveletLHL_glcm_ClusterProminence	8314	8799	4872	7328	1697	23349
N_T1w_waveletHLL_firstorder_Median	0.020	0.060	0.009	0.037	−0.026	0.068
N_T1w_waveletHLL_glrlm_GrayLevelVariance	18.758	12.089	14.600	14.020	7.841	34.622
N_T1w_waveletHHH_glcm_ClusterProminence	2884	3789	1429	2422	565	5996
N_T1w_waveletLLL_firstorder_RobustMeanAbsoluteDeviation	0.434	0.249	0.381	0.354	0.170	0.844
N_T1w_waveletLLL_firstorder_TotalEnergy	109302	192481	53891	105783	12483	244494
N_T2w_original_firstorder_Mean	1.456	0.474	1.398	0.522	0.939	1.957
N_T2w_original_firstorder_Skewness	−0.052	0.633	−0.016	0.974	−0.822	0.682
N_T2w_original_glcm_JointEntropy	7.152	0.622	7.230	0.876	6.295	7.918
N_T2w_original_glrlm_GrayLevelVariance	25.336	9.424	23.501	13.925	14.058	37.478
N_T2w_original_glrlm_LongRunHighGrayLevelEmphasis	445.905	139.035	425.249	168.643	299.069	603.548
N_T2w_waveletLLH_firstorder_Mean	−0.020	0.031	−0.017	0.023	−0.042	0.004
N_T2w_waveletLLH_firstorder_Median	−0.011	0.026	−0.009	0.023	−0.034	0.015
N_T2w_waveletLLH_glcm_ClusterProminence	9501	10282	5487	8288	2071	21430
N_T2w_waveletLLH_glcm_Imc1	−0.172	0.117	−0.132	0.115	−0.327	−0.076
N_T2w_waveletLLH_glcm_JointEnergy	0.033	0.047	0.014	0.017	0.007	0.109
N_T2w_waveletLHL_glcm_JointEntropy	7.031	0.668	7.087	0.877	6.190	7.734
N_T2w_waveletHHL_glcm_InverseVariance	0.265	0.068	0.268	0.084	0.177	0.361
N_T2w_waveletHHH_firstorder_Entropy	3.654	0.411	3.662	0.589	3.126	4.170
N_T2w_waveletLLL_firstorder_MeanAbsoluteDeviation	1.059	0.394	0.993	0.369	0.653	1.577
N_T2w_waveletLLL_firstorder_Range	9.235	3.258	8.696	3.859	5.914	13.607
N_T2w_waveletLLL_glcm_DifferenceAverage	2.870	1.022	2.623	1.302	1.772	4.351
N_T2w_waveletLLL_glcm_SumAverage	33.866	5.391	33.631	7.210	27.364	40.828

Values are displayed before Z-score normalization.

Table S2. Descriptive statistics of the 67 stable and non-redundant features used in the study.

**Stable_Feat_Names**	**Median Values Norm**	**Iqr Values Norm**	**Perc10 Values Norm**	**Perc90 Values Norm**
T_T1w_original_shape_Flatness	−0.204	1.409	−1.133	1.336
T_T1w_original_shape_MajorAxisLength	−0.071	1.092	−1.150	1.197
T_T1w_original_glcm_InverseVariance	0.174	1.084	−1.571	1.149
T_T1w_waveletLLH_firstorder_Median	0.151	1.016	−1.209	0.918
T_T1w_waveletLLH_glcm_JointEntropy	0.019	1.385	−1.204	1.252
T_T1w_waveletLHL_firstorder_10Percentile	0.191	1.122	−1.521	1.079

T_T1w_waveletLHL_firstorder_90Percentile	−0.121	1.271	−1.334	1.327
T_T1w_waveletLHL_firstorder_Median	0.278	0.890	−1.277	0.799
T_T1w_waveletLHL_glcm_SumEntropy	−0.014	1.531	−1.234	1.299
T_T1w_waveletHLL_firstorder_Median	0.002	0.710	−0.838	0.642
T_T1w_waveletHLL_glcm_DifferenceEntropy	−0.047	1.344	−1.163	1.438
T_T1w_waveletHHL_glcm_SumSquares	−0.202	0.976	−0.954	1.124
T_T1w_waveletHHH_glcm_ClusterProminence	−0.337	0.814	−0.768	1.321
T_T1w_waveletLLL_firstorder_Mean	−0.090	1.141	−1.012	0.999
T_T1w_waveletLLL_firstorder_RobustMeanAbsoluteDeviation	−0.117	1.208	−1.055	1.049
T_T1w_waveletLLL_firstorder_TotalEnergy	−0.317	0.729	−0.682	0.952
T_T2w_original_shape_Elongation	−0.026	1.572	−1.272	1.352
T_T2w_original_shape_MinorAxisLength	−0.038	1.294	−1.177	1.278
T_T2w_original_firstorder_90Percentile	−0.070	1.149	−1.079	1.148
T_T2w_original_firstorder_MeanAbsoluteDeviation	−0.100	1.334	−1.222	1.361
T_T2w_original_glcm_MaximumProbability	−0.341	1.419	−1.013	1.554
T_T2w_original_glcm_SumEntropy	0.059	1.450	−1.339	1.379
T_T2w_original_glrlm_LongRunHighGrayLevelEmphasis	−0.037	1.414	−1.129	1.344
T_T2w_waveletLLH_firstorder_Mean	0.027	1.403	−1.112	1.159
T_T2w_waveletLLH_glcm_ClusterProminence	−0.298	1.194	−0.966	1.314
T_T2w_waveletLLH_glcm_Imc1	0.178	0.799	−1.092	0.815
T_T2w_waveletLLH_glcm_MaximumProbability	−0.307	1.145	−0.895	1.305
T_T2w_waveletLHL_glcm_JointEntropy	0.161	1.556	−1.407	1.200
T_T2w_waveletHLL_glcm_Imc1	0.250	0.683	−1.164	0.731
T_T2w_waveletHHL_glcm_Idm	0.026	1.472	−1.276	1.131
T_T2w_waveletHHH_firstorder_Entropy	−0.036	1.464	−1.104	1.354
T_T2w_waveletLLL_firstorder_Range	−0.039	1.525	−1.236	1.336
T_T2w_waveletLLL_glcm_ClusterShade	0.037	1.184	−1.298	1.258
N_T1w_original_shape_Elongation	−0.027	1.369	−1.189	1.364
N_T1w_original_shape_Flatness	−0.107	1.270	−1.226	1.362
N_T1w_original_shape_Maximum2DDiameterColumn	−0.008	1.461	−1.362	1.144
N_T1w_original_firstorder_Median	0.005	1.179	−1.190	1.023
N_T1w_original_glcm_InverseVariance	0.309	1.219	−1.747	0.995
N_T1w_waveletLLH_firstorder_Mean	−0.069	0.856	−0.859	1.260
N_T1w_waveletLLH_firstorder_Median	−0.236	0.878	−0.833	1.270
N_T1w_waveletLLH_glcm_ClusterProminence	−0.361	0.903	−0.761	1.692
N_T1w_waveletLLH_glcm_JointEntropy	0.193	1.290	−1.621	1.009
N_T1w_waveletLHL_firstorder_10Percentile	0.284	1.492	−1.384	1.066
N_T1w_waveletLHL_firstorder_Median	−0.261	0.687	−0.650	1.101
N_T1w_waveletLHL_glcm_ClusterProminence	−0.391	0.833	−0.752	1.709
N_T1w_waveletHLL_firstorder_Median	−0.183	0.616	−0.762	0.807
N_T1w_waveletHLL_glrlm_GrayLevelVariance	−0.344	1.160	−0.903	1.312
N_T1w_waveletHHH_glcm_ClusterProminence	−0.384	0.639	−0.612	0.821
N_T1w_waveletLLL_firstorder_RobustMeanAbsoluteDeviation	−0.213	1.423	−1.060	1.645
N_T1w_waveletLLL_firstorder_TotalEnergy	−0.288	0.550	−0.503	0.702
N_T2w_original_firstorder_Mean	−0.123	1.103	−1.093	1.056
N_T2w_original_firstorder_Skewness	0.057	1.538	−1.215	1.160
N_T2w_original_glcm_JointEntropy	0.125	1.409	−1.379	1.231
N_T2w_original_glrlm_GrayLevelVariance	−0.195	1.478	−1.197	1.288
N_T2w_original_glrlm_LongRunHighGrayLevelEmphasis	−0.149	1.213	−1.056	1.134
N_T2w_waveletLLH_firstorder_Mean	0.085	0.739	−0.718	0.776
N_T2w_waveletLLH_firstorder_Median	0.092	0.881	−0.912	1.016
N_T2w_waveletLLH_glcm_ClusterProminence	−0.390	0.806	−0.723	1.160
N_T2w_waveletLLH_glcm_Imc1	0.335	0.981	−1.323	0.814
N_T2w_waveletLLH_glcm_JointEnergy	−0.406	0.367	−0.556	1.636
N_T2w_waveletLHL_glcm_JointEntropy	0.084	1.313	−1.259	1.053
N_T2w_waveletHHL_glcm_InverseVariance	0.035	1.243	−1.313	1.410
N_T2w_waveletHHH_firstorder_Entropy	0.020	1.433	−1.284	1.257

N_T2w_waveletLLL_firstorder_MeanAbsoluteDeviation	−0.167	0.938	−1.031	1.317
N_T2w_waveletLLL_firstorder_Range	−0.165	1.185	−1.020	1.342
N_T2w_waveletLLL_glcm_DifferenceAverage	−0.241	1.274	−1.074	1.450
N_T2w_waveletLLL_glcm_SumAverage	−0.044	1.338	−1.206	1.292

Values are displayed after Z-score normalization, and therefore mean and standard deviations are 0 and 1 respectively for all the features.

Table S3. List of stable and non-redundant features ordered by the number of times they were selected within the 100 bootstrap iterations.

Features Names	Number Of Selections
T_T1w_waveletLLH_firstorder_Median	39
T_T1w_waveletLLL_firstorder_Mean	23
T_T2w_original_firstorder_90Percentile	18
N_T1w_original_firstorder_Median	17
N_T2w_waveletLLH_firstorder_Mean	17
N_T2w_original_firstorder_Mean	14
T_T1w_original_glcm_InverseVariance	10
T_T1w_waveletLHL_firstorder_Median	10
T_T1w_waveletHLL_firstorder_Median	7
T_T2w_waveletLLH_firstorder_Mean	5
N_T2w_waveletLLH_glcm_JointEnergy	5
N_T2w_waveletHHH_firstorder_Entropy	5
T_T1w_waveletLHL_firstorder_10Percentile	4
T_T2w_original_firstorder_MeanAbsoluteDeviation	4
N_T2w_waveletLLH_firstorder_Median	4
T_T1w_waveletLHL_glcm_SumEntropy	3
T_T2w_waveletLLH_glcm_Imc1	3
N_T2w_waveletLLL_firstorder_Range	3
N_T1w_original_shape_Flatness	2
T_T1w_original_shape_MajorAxisLength	1
T_T1w_waveletHHH_glcm_ClusterProminence	1
T_T1w_waveletLLL_firstorder_RobustMeanAbsoluteDeviation	1
N_T1w_waveletLHL_glcm_ClusterProminence	1
N_T2w_waveletLLH_glcm_Imc1	1
N_T2w_waveletLHL_glcm_JointEntropy	1
N_T2w_waveletLLL_firstorder_MeanAbsoluteDeviation	1
T_T1w_original_shape_Flatness	0
T_T1w_waveletLLH_glcm_JointEntropy	0
T_T1w_waveletLHL_firstorder_90Percentile	0
T_T1w_waveletHLL_glcm_DifferenceEntropy	0
T_T1w_waveletHHL_glcm_SumSquares	0
T_T1w_waveletLLL_firstorder_TotalEnergy	0
T_T2w_original_shape_Elongation	0
T_T2w_original_shape_MinorAxisLength	0
T_T2w_original_glcm_MaximumProbability	0
T_T2w_original_glcm_SumEntropy	0
T_T2w_original_glrlm_LongRunHighGrayLevelEmphasis	0
T_T2w_waveletLLH_glcm_ClusterProminence	0
T_T2w_waveletLLH_glcm_MaximumProbability	0
T_T2w_waveletLHL_glcm_JointEntropy	0
T_T2w_waveletHLL_glcm_Imc1	0
T_T2w_waveletHHL_glcm_Idm	0
T_T2w_waveletHHH_firstorder_Entropy	0
T_T2w_waveletLLL_firstorder_Range	0
T_T2w_waveletLLL_glcm_ClusterShade	0
N_T1w_original_shape_Elongation	0
N_T1w_original_shape_Maximum2DDiameterColumn	0
N_T1w_original_glcm_InverseVariance	0
N_T1w_waveletLLH_firstorder_Mean	0
N_T1w_waveletLLH_firstorder_Median	0

N_T1w_waveletLLH_glcm_ClusterProminence	0
N_T1w_waveletLLH_glcm_JointEntropy	0
N_T1w_waveletLHL_firstorder_10Percentile	0
N_T1w_waveletLHL_firstorder_Median	0
N_T1w_waveletHLL_firstorder_Median	0
N_T1w_waveletHLL_glrlm_GrayLevelVariance	0
N_T1w_waveletHHH_glcm_ClusterProminence	0
N_T1w_waveletLLL_firstorder_RobustMeanAbsoluteDeviation	0
N_T1w_waveletLLL_firstorder_TotalEnergy	0
N_T2w_original_firstorder_Skewness	0
N_T2w_original_glcm_JointEntropy	0
N_T2w_original_glrlm_GrayLevelVariance	0
N_T2w_original_glrlm_LongRunHighGrayLevelEmphasis	0
N_T2w_waveletLLH_glcm_ClusterProminence	0
N_T2w_waveletHHL_glcm_InverseVariance	0
N_T2w_waveletLLL_glcm_DifferenceAverage	0
N_T2w_waveletLLL_glcm_SumAverage	0

Table S4. Descriptive statistics and Cox coefficients for the two volumes of interest (main tumor and largest lymphnode).

VOLUME FEATURES STATISTICS		
Feature	Tumor Volume	Largest Lymphnode Volume
Mean[1] (before/after normalization)	22169/0	16107/0
Standard deviation (before/after normalization)	17469/1	14298/1
Median[1] (before/after normalization)	16032/−0.351	11448/−0.326
Interquartile range[1] (before/after normalization)	19 668/1.126	17716/1.239
10th percentile[1] (before/after normalization)	5059/−0.979	1619/−1.013
90th percentile[1] (before/after normalization)	49165/1.545	37213/−1.476
Cox coefficient[1]	1.21×10^{-5}	1.94×10^{-5}

Volumes are expressed in mm^3.

Table S5. Radiomic features by iteration of the 10-fold cross validation.

MODELS BY ITERATION			
Iteration Number	Selected Features	Regression Coefficients	Signature Threshold
1	*- T-T1w-waveletLLH-firstorder-Median*<br>*- T-T1w-waveletHLL-firstorder-Median*	0.79<br>0.61	0.15
2	*- T-T1w-original-firstorder-Mean*<br>*- T-T1w-waveletLLH-firstorder-Median*	−0.72<br>1.07	0.23
3	*- N-T1w-original-firstorder-Median*<br>*- N-T2w-original-firstorder-Median*	−0.40<br>−0.67	0.06
4	*- T-T1w-waveletLLH-firstorder-Median*<br>*- N-T1w-original-firstorder-Median*	0.98<br>−0.67	0.21
5	*- T-T1w-original-firstorder-Mean*<br>*- T-T1w-waveletLLH-firstorder-Median*	−0.97<br>1.05	0.26
6	*- T-T1w-waveletLLH-firstorder-Median*<br>*- N-T2w-original-firstorder-Median*	0.99<br>−1.02	0.08
7	*- T-T1w-waveletLLH-firstorder-Median*<br>*- N-T2w-original-firstorder-Median*	0.95<br>−0.60	0.22
8	*- T-T1w-waveletLLH-firstorder-Median*<br>*- T-T1w-waveletLLL-firstorder-Mean*	1.26<br>−0.85	0.37
9	*- T-T1w-original-glcm-InverseVariance*<br>*- T-T1w-waveletLLH-firstorder-Median*	0.71<br>0.77	0.37
10	*- T-T1w-waveletLLH-firstorder-Median*<br>*- T-T2w-original-firstorder–90Percentile*	1.22<br>−0.85	0.11

For each iteration, the model is characterized by selected features, the corresponding Cox regression coefficients and the signature threshold used for the classification of the patients in high/low risk groups.

Table S6. Quality metrics with 95% confidence intervals for the two-volumes model (tumor and main lymphnode) for the different endpoint of interest.

**PERFORMANCE METRICS (TWO-VOLUMES MODEL)**				
Endpoint	Overall Survival	Disease-Free Survival	Locoregional Recurrence-Free Survival	Distant Metastasis-Free Survival
Hazard ratio (signature)	1.29 (0.30–5.56)	1.42 (0.54–3.73)	0.54 (0.10–2.77)	2.59 (0.77–8.76)
C-index (signature)	0.56 (0.44–0.67)	0.55 (0.45–0.64)	0.54 (0.41–0.67)	0.6 (0.47–73)
Hazard ratio (high vs low risk)	1.22 (0.46–3.29)	1.03 (0.54–1.96)	1.13 (0.44–2.95)	0.94 (0.39–2.25)

© 2020 by the authors. Licensee MDPI, Basel, Switzerland. This article is an open access article distributed under the terms and conditions of the Creative Commons Attribution (CC BY) license (http://creativecommons.org/licenses/by/4.0/).
